# RNA-seq dataset of superworm (*Zophobas atratus*) feeding on polystyrene foam

**DOI:** 10.1016/j.dib.2026.113053

**Published:** 2026-07-10

**Authors:** Yuh Shiwa, Shinya Kudo, Ayano Watanabe, Takeshi Doi, Mariko Shimizu-Kadota

**Affiliations:** a Department of Molecular Microbiology, Tokyo University of Agriculture, Sakuragaoka, Setagaya-ku, Tokyo, 156-8502, Japan; b NODAI Genome Research Center, Tokyo University of Agriculture, Setagaya-ku, Tokyo, 156-8502, Japan; c Department of Environmental Systems Sciences, Musashino University, Kōtō-ku, Tokyo 135-8181, Japan; d Musashino University Creating Happiness Incubation, Musashino University, Kōtō-ku, Tokyo 135-8181, Japan

**Keywords:** Insect, Transcriptome, Plastic, Degradation, Digestive enzymes, Environmental pollution

## Abstract

Superworms, the larvae of *Zophobas atratus* Fabricius 1775 (also known as *Zophobas morio* Fabricius 1776), can not only survive on a diet consisting solely of polystyrene (PS) but also degrade it. This degradation is thought to be a collaborative process involving enzymes from both the gut microbiome and the host insect. Although the effects of a PS diet on the gut microbiome of superworms have been studied, few studies have focused on the changes in host gene expression. This RNA-seq dataset was collected to investigate gene expression in three parts of superworms (head, gut, and leg) reared in three groups with wheat bran, PS, and starvation diets. Total RNA was extracted from the heads, guts, and legs of 24 superworms subjected to wheat bran, PS, or starvation conditions. Strand-specific RNA sequencing was performed after rRNA depletion using the NextSeq 1000 PE150 platform. The CLC Genomic Workbench was used for reference-based RNA-seq analysis. We also used rnaSPAdes to assemble unmapped reads that could not be aligned to the reference genome to generate contigs, including genes expressed in the microbiome. These RNA-seq datasets provide a valuable resource for identifying genes encoding PS-degrading enzymes involved in various metabolic pathways in superworms.

Specifications Table

**Table:** 

Subject	Biology
Specific subject area	Omics: RNA-seq analysis of polystyrene-feeding superworm (larva of *Zophobas atratus*)
Type of data	Raw sequence (FASTQ), Tables, Figures, and Assembled contigs (FASTA)
Data collection	Total RNA was extracted from the heads, guts, and legs of 24 superworms reared in three feeding conditions: wheat bran, polystyrene (PS), and starvation. Strand-specific RNA sequencing following rRNA depletion was performed using the NextSeq 1000 PE150 platform. CLC Genomic Workbench v23.0.5 was used for the reference-based RNA-seq analysis. We also used rnaSPAdes v4.2.0 to assemble unmapped reads that could not be mapped to the reference genome to generate contigs that may include genes expressed by symbiotic microbes.
Data source location	The data were collected and analyzed at the Tokyo University of Agriculture, Tokyo, Japan. Superworms were purchased from a Fish Japan pet shop (Tokyo, Japan).
Data accessibility	Repository name: DNA Data Bank of Japan (DDBJ), DDBJ Sequence Read Archive (DRA) and in the Zendo data repository. Data identification number: PRJDB42187 (BioProject) Direct URL to data: The sequencing data were deposited in the Japan DNA Data Bank (DDBJ) and the DDBJ Sequence Read Archive (DRA) and can be accessed using the BioProject ID listed above (https://ddbj.nig.ac.jp/search/entry/bioproject/PRJDB42187). The assembled nucleotide sequences in FASTA format are publicly available in Zenodo at https://zenodo.org/records/20177339 (DOI: 10.5281/zenodo.20177338).
Related research article	None

## 1 Value of the Data

• RNA-seq data provide a valuable resource for investigating the mechanisms underlying polystyrene (PS) degradation in superworms (*Zophobas atratus* larvae).
• Using the latest and most accurate reference genome, RNA-seq analysis enables the quantification of gene expression levels at specific genomic loci.
• The gene expression table (Table S2) includes valuable annotations, including Gene Ontology, Kyoto Encyclopedia of Genes and Genomes Orthology, and Carbohydrate-Active Enzyme annotations.

## 2 Background

Humans produce and dispose of large amounts of plastic. These polymers are difficult for microbiota to decompose in the natural environment and pollute oceans and land, threatening the survival of the organisms that live there. Biodegradation by insect larvae has been the focus of active research [1]. Superworms, the larvae of *Zophobas atratus* Fabricius 1775 (also known as *Zophobas morio* Fabricius 1776) [2,3], can not only survive on a diet consisting solely of polystyrene (PS) but also metabolize it [4]. This is thought to be a cooperative process involving enzymes derived from both gut bacteria and the host insects [5].

Although the effects of a PS diet on the gut microbiome of superworms have been studied [6], few studies have focused on the changes in gene expression in host superworms. However, it has been reported that digestive enzymes derived from the saliva of waxworms (larvae of *Galleria mellonella*) can degrade polyethylene [7]. Enzymes other than those in the gut may also be involved in the degradation of PS in superworms.

The present dataset was collected to investigate gene expression in three parts of superworms (head, gut, and leg) reared under three feeding conditions: wheat bran, PS, and under starvation conditions.

## 3 Data Description

### 3.1 Raw data

These data describe the RNA-seq experiments conducted on superworms reared under three different feeding conditions (wheat bran, PS, and starvation) and originated from three different body parts (head, gut, and leg). Based on RNA concentration and quality (RINe value: 8.9 ± 0.2), 10 head samples, 11 gut samples, and 6 leg samples were selected for RNA-seq. Strand-specific RNA-seq was performed after rRNA depletion using the NextSeq 1000 PE150 platform, which yielded FASTQ files for each sample. Sequencing generated an average of 18 ± 1.6 million paired-end reads per sample (Supplementary Table 1).

### 3.2 Mapping to the *Z. atratus* reference genome

The FASTQ files obtained were imported into the CLC Genomics Workbench, where low-quality reads were trimmed. Quality-controlled reads were mapped to CSIRO_AGI_Zmor_V1 (GCF_036711695.1) in NCBI Genomes (https://www.ncbi.nlm.nih.gov/home/genomes/) as the representative reference genome for *Z. morio* (*atratus*) at the time of analysis. The average mapping rate was 82.1 ± 1.9%, with gut-derived reads showing a lower mapping rate than head- and leg-derived reads (Supplementary Table 1).

### 3.3 Transcriptome profiling

**Fig. 1:**
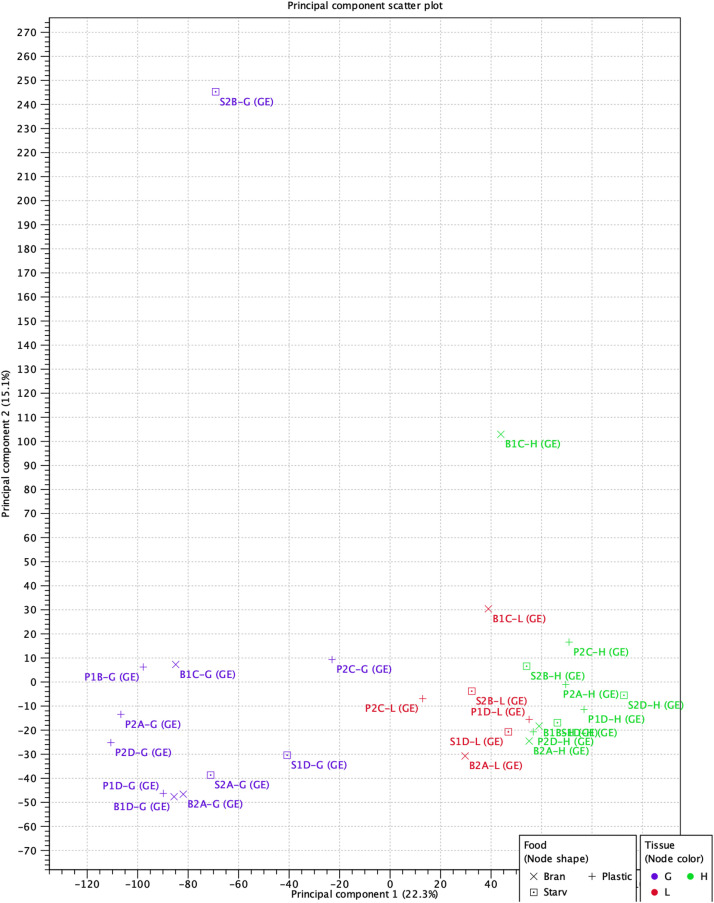
PCA score plot of RNA-seq samples. Each point represents a sample and is colored by part type (G: gut, H: head, and L: leg) and shaped by food source (bran, starvation, or plastic (PS)). Sample names followed by (GE) indicate that the analysis was performed based on gene-level expression profiles. The percentages on the axes indicate the proportion of variance explained by each of the principal components.

**Fig. 2:**
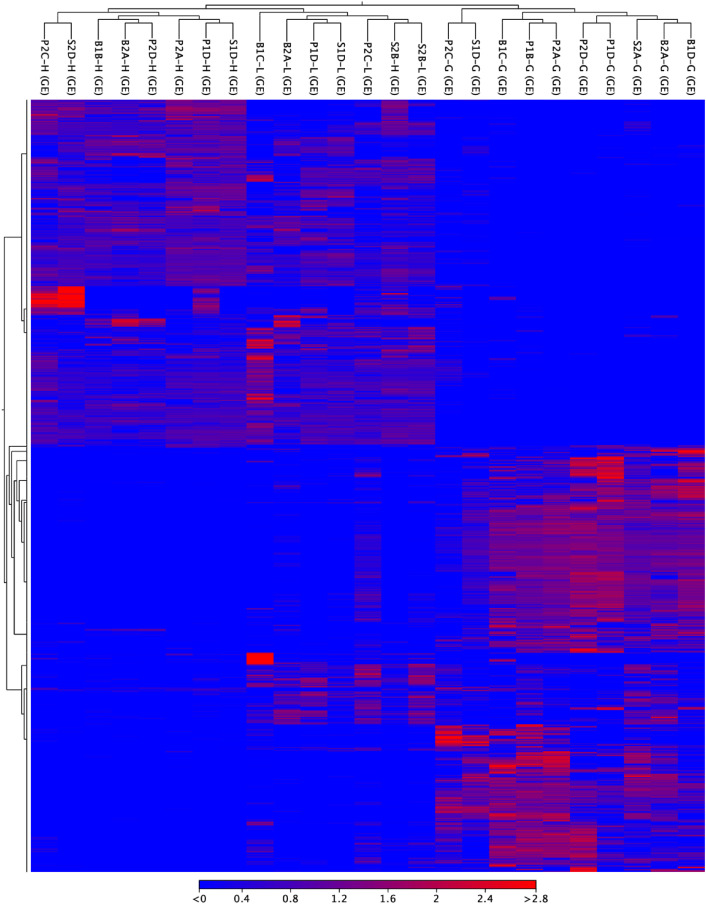
Hierarchical clustering and heatmap of gene expression across samples. The heatmap shows the expression profiles of the 7962 genes in different part types and under various dietary conditions (see Transcriptome Analysis section of EXPERIMENTAL DESIGN, MATERIALS AND METHODS). Rows represent individual genes and columns represent biological samples. The color scale at the bottom represents normalized expression levels (log2 CPM (counts per million)), with red and blue indicating higher and lower expression, respectively. Sample IDs followed the format: [Sample ID]-[Part Type] (G: gut, H: head, or L: leg).

To assess the overall similarity and variance among the samples, Principal Component Analysis was performed based on the gene expression profiles (Fig. 1). Samples were clustered according to body part. However, two samples (S2B-G and B1C—H) were excluded from subsequent analyses because they were outliers. Next, we identified the part-specific differentially expressed genes (DEGs). These genes were identified by filtering the value of the “Due to Tissue - FDR p-value” column in Supplementary Table 2 to <0.05. A heatmap was generated using the expression levels of 7962 genes (Fig. 2). The dendrogram of the heatmap revealed three part-specific clusters, except for one sample (S2B-H).

### 3.4 DEG analysis

We analyzed the DEGs among all 27,339 genes in the reference genome (Supplementary Table 2). We conducted intergroup comparisons between body parts and diets. Because the reference genome annotation contained limited functional information, we used the eggNOG-mapper to assign Gene Ontology, Kyoto Encyclopedia of Genes and Genomes Orthology, and Carbohydrate-Active Enzyme annotations to each gene.

**Fig. 3:**
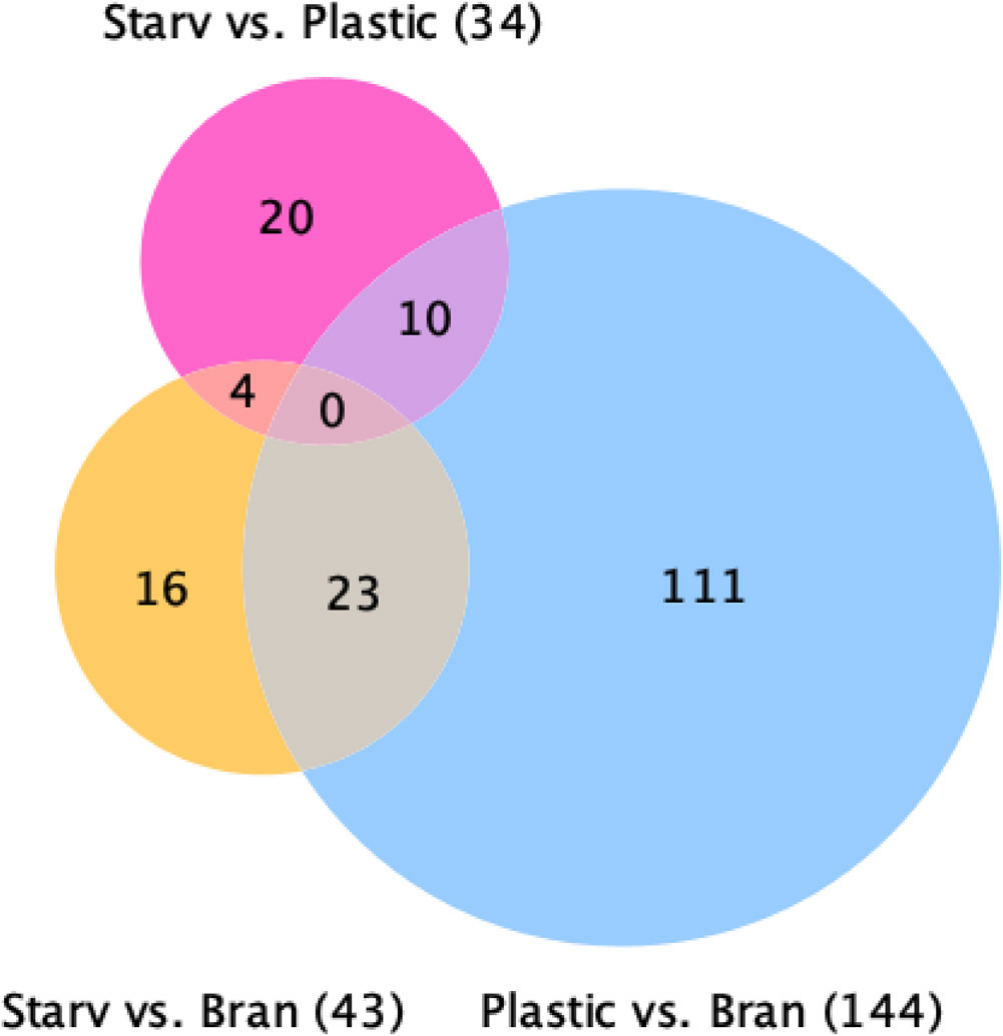
Identification of DEGs across dietary conditions. The Venn diagram illustrates the number of unique and shared DEGs identified in the gut among three pairwise comparisons: Starvation vs. Plastic (PS), Starvation vs. Bran, and Plastic (PS) vs. Bran. DEGs were defined based on a minimum absolute fold change of 2 and a False Discovery Rate (FDR) of < 0.05.

As enzymes derived from the gut are expected to be involved in PS degradation, the Venn diagram in Fig. 3 shows the number of DEGs in the gut across feeding groups. For example, differences in the expression levels of 144 genes were observed between the PS (plastic) and wheat bran groups. Of these, the expression levels of 64 genes were more than two-fold in the PS group than those in the Bran group. These genes were identified by filtering the values shown in Supplementary Table 2: genes with “G-Plastic vs. Bran - Log fold change” ≥ 1 and “G-Plastic vs. Bran - FDR p-value” values <0.05. Similarly, we identified genes whose expression varied across feeding conditions by filtering the “Log fold change” and “FDR p-value” columns.

### 3.5 *de novo* transcriptome assembly of unmapped reads

Since these data were generated from rRNA-depleted RNA sequencing, sequences that did not map to the reference genome of the insect also included enzyme gene sequences derived from the insect microbiota. We obtained these sequences by performing *de novo* transcriptome assembly using reads that did not map to the reference genome (an average of 12.6 ± 2.0% of reads per sample). This assembly yielded 505,217 transcripts, including 279,293 transcripts that were ≥500 bp and 191,380 that were ≥1 kb in length. The assembled nucleotide sequences in FASTA format are publicly available in Zenodo at https://zenodo.org/records/20177339 (DOI: 10.5281/zenodo.20177338).

## 4 Experimental Design, Materials and Methods

### 4.1 Feeding trials with superworms

Superworms, larvae of *Z. atratus,* were purchased online from the Fish Japan pet shop (Tokyo, Japan). Upon arrival, approximately 70 large larvae were visually selected and fed wheat bran at room temperature for 15 days during the acclimatization period.

After the acclimation period, the 60 heaviest larvae were selected and divided into six groups of 10 larvae each to ensure equivalent average weights (average body weight, 0.098 g). Each group was placed in a 200 mL glass beaker and reared for 22 days under three conditions. (i) The two bran groups: fed wheat bran; (ii) the two polystyrene (PS) groups: fed Styrofoam blocks and distilled water; and (iii) the two starved groups: fed only distilled water. Styrofoam blocks made of PS, purchased from Seiun-shouten (Osaka, Japan) and cut into pieces measuring 5 cm × 5 cm × 1.5 cm, were placed inside glass beakers. Body weight was measured weekly, and feeding and fecal removal were performed at the end of each week. All the experiments were conducted at room temperature without humidity control.

### 4.2 Weight measurement and dissection

After rearing, four large individuals were visually selected from each group and their body weights were measured (three rearing conditions × two replicates × four individuals = 24 individuals). The average body weights for each condition were 0.126 ± 0.005 g for the bran group, 0.113 ± 0.001 g for the PS group, and 0.117 ± 0.003 g for the starvation group, with one-way ANOVA showing no statistically significant differences between the groups (p = 0.096). The fact that no significant decrease in biomass was observed in the starvation group and that the average body weight was higher than that at the start of the experiment (0.098 g) is thought to be attributable to the group-rearing strategy. The larvae were reared in groups of 10 to prevent pupation due to isolation; however, this high-density environment under nutrient-deficient conditions led to cannibalism. This behavior may have influenced the average body weight of the surviving individuals at the final measurement. After weighing, three or four of the heaviest larvae from each group were selected, yielding a total of 13 larvae. The larvae were frozen in liquid nitrogen and immediately dissected into head, leg, gut, and other body parts. The dissected parts were placed in tubes containing 200 µL of DNA/RNA Shield (Zymo Research, USA) and stored at −80 °C.

### 4.3 RNA extraction

RNA was extracted from the samples (head, gut, and leg) of single larvae and homogenized using BioMasher II and PowerMasher II (Nippi, Japan). Subsequently, RNA was extracted from the samples using Maxwell RSC simplyRNA cells and simplyRNA tissue kits (Promega, USA) according to the manufacturer’s protocols. The RNA concentration and purity were measured using a DeNovix DS-11 spectrophotometer (DeNovix, Wilmington, DE, USA). RNA integrity was assessed using an RNA ScreenTape on an Agilent 2200 TapeStation (Agilent Technologies, USA), and the RNA concentration values obtained from the TapeStation were used for subsequent analyses.

### 4.4 cDNA library and Illumina sequencing

High-quality RNA samples (RINe greater than 7.0) were selected for library preparation. Libraries were constructed from 250 ng of RNA using the Zymo-Seq RiboFree Total RNA Library Preparation Kit (Zymo Research, USA) according to the manufacturer’s protocol. Library quality was assessed using a High Sensitivity D1000 ScreenTape on an Agilent 2200 TapeStation (Agilent Technologies, USA), and pooled libraries were quantified using KAPA Library Quantification Kits for Illumina platforms, following the qPCR protocol guide. The indexed libraries were subsequently sequenced on an Illumina NextSeq 1000 platform using paired-end sequencing (2 × 150 bp).

### 4.5 Transcriptome analysis

Raw data (FASTQ files) were analyzed using the CLC Genomics Workbench v23.0.5 (QIAGEN). Raw paired-end reads were imported into the software, and quality control was performed to remove low-quality reads and adapter sequences. High-quality reads were mapped to the *Z. morio* (*atratus*) isolate AGI-343–1 reference genome (CSIRO_AGI_Zmor_V1; GCF_036711695.1) using the default parameters of the RNA-Seq Analysis module. The average gene expression values normalized to transcripts per million are presented in Supplementary Table 2. Using statistical tools built into the CLC Genomics Workbench, we generated heat maps, analyzed the differentially expressed genes, and created Venn diagrams. Part-specific gene expression was determined using a built-in ANOVA-like test. Protein sequences included in the reference genome were further annotated using eggNOG-mapper v2.1.13 [8] to assign functional categories and obtain orthologous information.

## Limitations

Because dissection of the larval head, gut, and leg was performed visually without the use of a microscope, homogeneity among biological replicates may have been compromised for each part. However, part-specific expression profiles were observed in the RNA-seq data. Furthermore, the number of biological replicates for leg samples in this study was limited (n = 2). Although this sample size may reduce the statistical power for the identification of DEGs, the design, which was optimized for high-depth sequencing, yielded a comprehensive spatial transcriptome profile. Therefore, this dataset should be considered a valuable pilot resource for identifying candidate genes for future large-scale validation studies.

## Ethics Statement

No specific ethical approval was required for this study as it involved only non-regulated invertebrate species. All experiments were performed in accordance with relevant institutional and national guidelines for the ethical treatment of insects.

## Declaration of Generative AI and AI-assisted Technologies in the Writing Process

During the preparation of this work, the authors used Google Gemini to improve language and readability. After using this tool/service, the authors reviewed and edited the content as required and took full responsibility for the publication.

## Credit Author Statement

**Yuh Shiwa:** Supervision, Methodology, Investigation, Data curation, Visualization, Writing, Original draft preparation. **Shinya Kudo:** Investigation. **Ayano Watanabe:** Investigation. **Takeshi Doi:** Conceptualization, Investigation**. Mariko Shimizu-Kadota:** Supervision, Funding acquisition, Project administration**,** Conceptualization, Methodology, Investigation, Writing - Review & Editing.

## Data Availability

DDBJ Sequence Read Archive (DRA)The sequencing data (FASTQ) (Original data)

## Appendix Supplementary Materials

Image, application 1

## References

[1] Yang S.S., Wu W.M., Bertocchini F., Benbow M.E., Devipriya S.P., Cha H.J., Peng B.Y., Ding M.Q., He L., Li M.X., Cui C.H., Shi S.N., Sun H.J., Pang J.W., He D., Zhang Y., Yang J., Hou D., Xing D.F., Ren N.Q., Ding J., Criddle C.S. (2024). Radical innovation breakthroughs of biodegradation of plastics by insects: history, present and future perspectives. Front. Env. Sci. Eng..

[2] Tschinkel W.R. (1981). Larval dispersal and cannibalism in a natural population of Zophobas atratus (Coleoptera: tenebrionidae). Anim. Behav..

[3] Rumbos C.I., Athanassiou C.G. (2021). The superworm, zophobas morio (Coleoptera:tenebrionidae): a ‘sleeping giant’ in nutrient sources. J. Insect Sci..

[4] Yang Y., Wang J., Xia M. (2020). Biodegradation and mineralization of polystyrene by plastic-eating superworms Zophobas atratus. Sci. Total Env..

[5] Kim H.R., Lee C., Shin H., Koh H.Y., Lee S., Choi D. (2024). Transcriptomic response of superworm in facilitating polyethylene biodegradation. J. Polym. Env..

[6] Sun J., Prabhu A., Aroney S.T.N., Rinke C. (2022). Insights into plastic biodegradation: community composition and functional capabilities of the superworm (Zophobas morio) microbiome in styrofoam feeding trials. Microb. Genom..

[7] Sanluis-Verdes A., Colomer-Vidal P., Rodriguez-Ventura F., Bello-Villarino M., Spinola-Amilibia M., Ruiz-Lopez E., Illanes-Vicioso R., Castroviejo P., Cigliano R.A., Montoya M., Falabella P., Pesquera C., Gonzalez-Legarreta L., Arias-Palomo E., Solà M., Torroba T., Arias C.F., Bertocchini F. (2022). Wax worm saliva and the enzymes therein are the key to polyethylene degradation by Galleria mellonella. Nat. Commun..

[8] Huerta-Cepas J., Forslund K., Coelho L.P., Szklarczyk D., Jensen L.J., Von Mering C., Bork P. (2017). Fast genome-wide functional annotation through orthology assignment by eggNOG-mapper. Mol. Biol. Evol..

